# Otology

**Published:** 1898-12

**Authors:** W. H. Harsant


### OTOLOGY.

Dr. Fred. Whiting,[1] of New York, has written an able and exhaustive article on the **symptomatology and treatment of sinus thrombosis.** He points out that sinus thrombosis is a disease of adult life, and occurs with greatest frequency between the ages of twenty and forty. Men, rather than women, are its victims in the proportion of 70 to 30. The right side is more

[1] *Arch. Otol.*, 1898, xxvii. 26.

commonly involved than the left, as might be expected from the structural peculiarities of the right temporal bone. Literature contains 132 cases of sinus thrombosis which have been operated upon; in addition to this number there have been reported at the New York Otological Society and elsewhere, but not as yet published by the operators, seven additional cases, making a total of 139. Of these, 95 terminated in recovery and 44 in death.

In an historical review of the literature of the subject, Whiting considers that Abercrombie was the first to call attention to thrombosis of the lateral sinus. This was in his work on *Diseases of the Brain*, published in 1829. But Dr. James Cowles Prichard, of Bristol, in his work on *Diseases of the Nervous System*, published in 1822, page 176, describes the case of a girl aged 16, who died in the Bristol Royal Infirmary after having had epileptic fits for two years, and at the *post-mortem* examination: "the left lateral sinus, through its whole length, was filled up by a substance, very different in its nature from a recent coagulum, and apparently consisting of a deposition of lymph, which had become organized. It appeared so completely to occupy the calibre of the sinus as to have entirely impeded the transit of blood through it." He does not state whether any old disease of the ear was found, but judging from his description of the symptoms there can be little doubt that such was the case, and that we owe the first description of this interesting affection to a Bristol physician.

In recent years the most notable writers upon the subject have been Macewen, Victor Horsley, Ballance and Lane in this country, and Zaufal, Körner, Hessler and Schwartze abroad.

The principal **local evidences** of the affection as observed by Whiting are: *pain*, usually radiating from the ear over the corresponding side of the head, varying in intensity from dull aching to violent cephalalgia of unendurable severity. *Œdema* of the mastoid region, extending backward and upward over the site of exit of the mastoid vein, and downward to that portion of the scalp drained by the occipital vein, is often present. Gerhardt has claimed that pressure over the external jugular vein would show that there was a decided diminution in the amount of blood passing through the vein of the affected side. *Tenderness* in the upper portion of the posterior cervical triangle, upon the importance of which Griesinger insists, is as often absent as present, but when it exists, is a valuable aid in estimating the probable position and extent of the obstructing thrombus. *Intra-ocular inflammatory changes* are observed in a considerable number of cases, and usually take the form of neuro-retinitis.

The **general or systemic symptoms** are essentially those of septicæmia. A feeling of malaise is preceded or followed by a sharp *chill* and a sudden and pronounced *rise in temperature*, 106° F. being frequently recorded. This marked pyrexia is subject to frequent remissions, and the amplitude of the exacerbation is at times very great, although the febrile period may be exceedingly brief, two hours sufficing in numerous instances for a variation of 6° F. Equally important is the appearance of *rigors*, which constitute a prominent feature at all stages of sinus thrombosis. They occur early, are frequently repeated, and as the toxæmia increases may even become daily manifestations, accompanied by profuse perspiration. It is true they may be entirely wanting, and 16 such cases are recorded, also 40 in which but a single chill was experienced, as against 200 in which the rigors were frequent. *Vertigo* is present in a moderate proportion of cases which are uncomplicated, and, like vomiting, is more constant when associated with meningitis. *Consciousness* is variable. In many cases, particularly if uncomplicated with meningitis, it remains unimpaired up to the moment of death. Loss of consciousness has been observed in 30 per cent. of uncomplicated cases, and in 50 per cent. of cases complicated with meningitis and brain abscess.

With the appearance of the foregoing symptoms there is usually *loss of appetite* and *constipation*, although later in the disease, as the septic influences become more pronounced, *diarrhœa* is almost uniformly present.

*Pulmonary manifestations* begin insidiously, usually with slight dyspnœa and cough, and patients complain of local areas of pain over the chest. These pains are followed in the course of twenty-four hours or so by rusty sputum and moist râles.

The *abdominal type* manifests itself in symptoms of a typhoid character. Septic enteritis has indeed been diagnosed under misapprehension of the conditions and failure to recognise the otorrhœa as the etiological factor.

The **meningeal group** of symptoms is less frequently encountered than either of the preceding, and is rarely found without association with one or other of the formerly mentioned groups; the meningitis may arise from the infective thrombosis, but it may also originate directly from the primary source of the disease and appear as a complication, the symptoms of which may predominate over those of the sinus thrombosis. *Strabismus* is a common symptom. In the later stages *delirium* supervenes, and the patient becomes comatose, after which the fatal termination is never long delayed.

The diagnosis in a typical case where the chronic suppuration of the ear is recognised, associated with repeated and severe chills, sudden and excessive rises of temperature, with rapid remissions, the establishment of metastases, either central or peripheral, and obstruction of the jugular, sufficiently pronounced to be recognisable to the touch, does not offer great difficulties. But it is highly essential to the successful prosecution of treatment that the condition be recognised if possible before the establishment of those symptoms constituting pyæmia, that is to say, in the early septic stages. Here the presence of Griesinger's symptoms, œdema of the region of the occipital vein with marked tenderness on pressure in the upper portion of the post-cervical triangle, will be a guide; and if the not thoroughly accepted Gerhardt symptom of diminished flow through the external jugular of the affected side can be determined, with rigors and sudden rises and remissions of temperature, with occasional vomiting and perhaps œdema of the eyelids of the affected side, with paresis of one or more nerves located in the region of the cavernous sinus, the diagnosis, if not assured, is at least sufficiently probable to justify the adoption of operative interference.

It is a disputed point whether it is necessary to ligature the internal jugular vein as an essential part of the operation for sinus thrombosis. Among those who practise **jugular ligation** are Zaufal, Victor Horsley, Lane, Ballance, and Macewen; while Schwartze and Salzer, with their students and followers, are chiefly those who do not countenance ligation. Schwartze's chief arguments against ligation are: that many cases have recovered without it; that tying one jugular does not guarantee that infective particles may not be carried into the system through the other; that the procedure adds materially to shock of operation, as the jugular is at times well-nigh impossible to find, owing to cellular infiltration of the neck; and that under certain circumstances fatal hemorrhage might ensue on opening the neck. The balance of opinion is however decidedly in favour of ligation as a necessary part of the operation. It is a safeguard against metastatic involvement of the lungs and other organs, and the additional shock to the patient incident upon such operation, when rapidly and skilfully performed, is not of sufficient moment, when weighed against the proportionately increased immunity thus afforded from general infection, to constitute a valid objection to the procedure.

Three successful operations are recorded by Whiting, nine cases with one death by Edward B. Dench, of New York,[1] and one successful case by J. E. Shephard, of Brooklyn.[2]

W. H. HARSANT.

---

### Reviews of Books.

**Religio Medici, and other Essays.** By Sir THOMAS BROWNE. Edited by D. LLOYD ROBERTS, M.D. Pp. xxxix., 305. London: Smith, Elder and Co. 1898.

However laudable a project it may be to attempt to revive the popularity of Sir Thomas Browne's writings, we confess that we are not very sanguine of its success. Dr. Lloyd Roberts has been at much pains to put forth a correct and handy edition of some of his works, or "pieces" as the author would have

[1] *Laryngoscope*, 1898, v. 104. [2] *Arch. Otol.*, 1898, xxvii. 357.

